# Chronic brucellosis: Recurrence in a calcified lesion of the hip

**DOI:** 10.1016/j.idcr.2026.e02829

**Published:** 2026-11-04

**Authors:** Christoph Zacher, Sebastian Dolff, Oliver Witzke, Lukas van de Sand

**Affiliations:** Department of Infectious Diseases, University Hospital Essen, University Duisburg-Essen, Essen, Germany

**Keywords:** Brucellosis, *Brucella melitensis*, Chronic infection

## Abstract

A patient with chronic brucellosis and ossifying granulomas was treated for several years with rifampicin, doxycycline and gentamicin. Despite repeated antibiotic treatment, local symptoms persisted, notably pain in the right hip joint and fistula formation. Imaging examinations showed stable calcified masses, while molecular polymerase chain reaction (PCR) tests finally remained negative. The clinical stability despite persistent symptoms indicates reduced bacterial activity in the presence of a chronic inflammatory reaction.

## Introduction

Brucellosis is a zoonotic infection caused by bacteria of the genus *Brucella*, which are Gram-negative, facultative intracellular coccobacilli. Despite being considered a neglected disease in many industrialized nations, brucellosis remains a significant public health concern in endemic regions. Globally, more than 500,000 new cases are reported each year, with actual numbers likely underestimated due to underdiagnosis and underreporting, especially in low-resource settings [1]. The primary modes of human transmission include the consumption of unpasteurized dairy products, direct contact with infected animals or animal products through injured skin or mucous membranes (e.g., conjunctivae), and inhalation of infectious aerosols—particularly among laboratory personnel [2]. Endemic areas include the Mediterranean basin, the Middle East, Africa, and parts of Central and South America [1]. Clinically, brucellosis is characterized by its protean manifestations and its potential for chronicity. The disease can affect multiple organ systems, often leading to diagnostic delays. Chronic forms may involve osteoarticular structures, with sacroiliitis and spondylitis being the most common complications [3]. Although the mortality rate of untreated brucellosis is generally low (1%), fatal outcomes may occur, primarily due to endocarditis, which is rare (< 2%) but remains one of the leading causes of brucellosis-related death [3], [4].

## Case presentation

The 22-year-old female patient, a refugee from Somalia with no known medical history, presented initially in a cardio-respiratory stable condition to a primary care hospital with lower abdominal pain. Clinically, she showed a soft abdomen without signs of peritonism, but with marked tenderness in the right lower quadrant. On palpation, a firm, fist-sized mass was detected in the right inguinal region. Additionally, there was a notable restriction in the mobility of the right leg. Initial laboratory investigations revealed only mildly elevated inflammatory markers, with a C-reactive protein (CRP) level of 3.3 mg/dL. Liver and renal function parameters were within normal limits. Notably, creatine kinase (CK) was markedly elevated at 1117 U/L, whereas creatine kinase-MB (CK-MB) and troponin levels remained within the normal range. A CT scan of the abdomen was performed and showed a bizarrely configured tumorous bone process with an irregular shape, measuring 15 × 8.7 × 10.4 cm and intrapelvic growth. The patient was referred to the university hospital for surgical biopsy and further treatment. Pathological examination of the specimen from the proximal right femur revealed extensive necrotic material and granulomatous inflammation with caseous necrosis. Therefore, an infectious cause was suspected and further microbiological examinations were performed. These yielded *Brucella melitensis* in the culture enrichment (Table 1).

**Table 1:** Longitudinal microbiological findings during the course of disease.

**Date**	**Clinical context**	**Specimen**	***Brucella spp.*****RT-PCR (IS711 and BCSP31)**	**Culture result**	**Antibiogram**	**Antibiotic therapy**
06/2018	Initial diagnosis	Proximal right femur	positive	*Brucella melitensis* isolated after enrichment culture	Gentamicin: S, Rifampicin: I, Streptomycin: S, Doxycycline: S	Doxycycline and Rifampicin
11/2021	Recurrence of B symptoms and new fistula formation	Right inguinal wound swab	positive	negative	n/a	Doxycycline and Rifampicin
02/2023	New fistula formation	Right labia majora wound swab and biopsy	negative	negative	n/a	Doxycycline and Rifampicin
07/2024	Follow-up before treatment interruption	Fistula secretion, swab	n/a	negative	n/a	none
01/2025	Surgical biopsy and revision	Intraoperative tissue sample	negative	negative	n/a	Doxycycline, Rifampicin and Gentamicin

In July 2018, the patient was hospitalised due to the confirmed diagnosis of brucellosis with ossifying lesions in order to initiate anti-infective therapy. The lesions involved the proximal right femur, the right iliac bone, and the adjacent intrapelvic soft tissues, forming a large mass with pelvic extension. Clinically, the patient presented with a secreting, but non-irritated biopsy wound. The initial treatment consisted of an oral combination of rifampicin and doxycycline as well as intravenous administration of gentamicin. During the initial phase of treatment, gentamicin was administered for 15 days without achieving adequate peak serum concentrations. Consequently, intravenous gentamicin therapy was extended to ensure a cumulative treatment period of 14 days with therapeutic drug levels, defined as peak concentrations of 5–10 µg/ml and trough concentrations < 1 µg/ml. The total duration of gentamicin therapy was therefore 29 days. During the course of treatment, the patient required multiple intravenous potassium substitutions due to recurrent hypokalemia. Secondary findings revealed a drug-induced toxic vestibulopathy, which was assessed as the cause of the new onset of vertigo. A magnetic resonance imaging (MRI) of the pelvis in August 2018 confirmed a stable, large-volume mass without significant changes (Fig. 1). After completing antibiotic treatment, the patient was discharged and scheduled for clinical follow-up at the outpatient clinic.

**Fig. 1:**
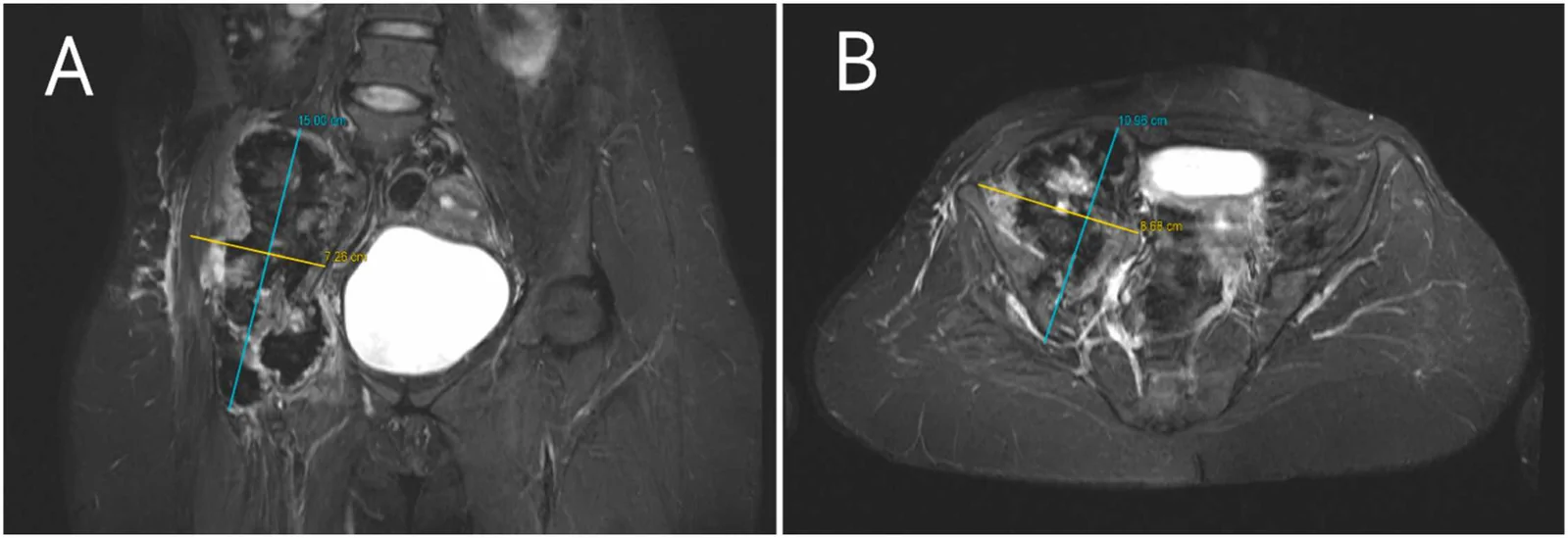
MRI of the pelvis from 20 August 2018. A predominantly calcified mass on the right inguinal/ iliac side and an unchanged infiltration of the right iliac bone can be seen, as well as borderline large inguinal lymph nodes. A. Coronal reconstruction, B. Axial reconstruction.

In February 2019, the patient complained of pain in her right hip joint; MRI imaging showed an unchanged mass. As the patient had already received prolonged combination therapy exceeding the treatment duration commonly recommended for osteoarticular brucellosis and no radiological progression was evident, oral doxycycline therapy was resumed as a suppressive treatment strategy during follow-up. In November 2021, systemic symptoms such as night sweats and a significant weight loss of 10 kg within two months occurred. In addition, insufficient adherence to doxycycline therapy was reported by the patient. She presented with a right inguinal fistula that promoted a putrid secretion. A swab from the right inguinal area showed a renewed positive result for *Brucella* in the real-time polymerase chain reaction (RT-PCR) test. Detection of *Brucella* DNA was performed targeting the insertion sequence IS711 and the bcsp31 gene, both established molecular markers for the detection of *Brucella* species. Intravenous gentamicin therapy was reinstituted for 14 days and oral rifampicin and doxycycline therapy was continued. PCR and microbiological culture tests remained negative in the subsequent outpatient follow-up visits.

In February 2023, the patient developed a new fistula tract in the right labia majora next to the pre-existing fistula in the right inguinal area. This gave rise to the suspect of a progression of the disease or the development of resistance under the current therapy. Follow-up laboratory investigations showed a mildly elevated C-reactive protein (CRP) level of 3.0 mg/dL, while all other laboratory parameters, including leukocyte count, liver function tests, and renal function tests, remained within normal limits. Material from wound swabs and skin punches were sent in for resistance testing. In July 2024, antibiotic treatment was temporarily paused after PCR tests for *Brucella spp.* were negative and only calcified granuloma residues were visible on imaging. In January 2025, the patient was admitted to hospital again for surgical sampling due to progressive secretion via both fistula tracts. Histopathology revealed a calcification with few cells, mild fibrosis and a low-grade inflammatory infiltrate, with no evidence of malignancy. The microbiological evaluation of the samples was still pending at this time. A new calculated therapy was subsequently initiated with rifampicin, doxycycline and gentamicin for four weeks. The February 2025 pelvic MRI revealed a partly calcified, mostly constant-size brucellosis presentation with growth in the right ileal os (Fig. 2).

**Fig. 2:**
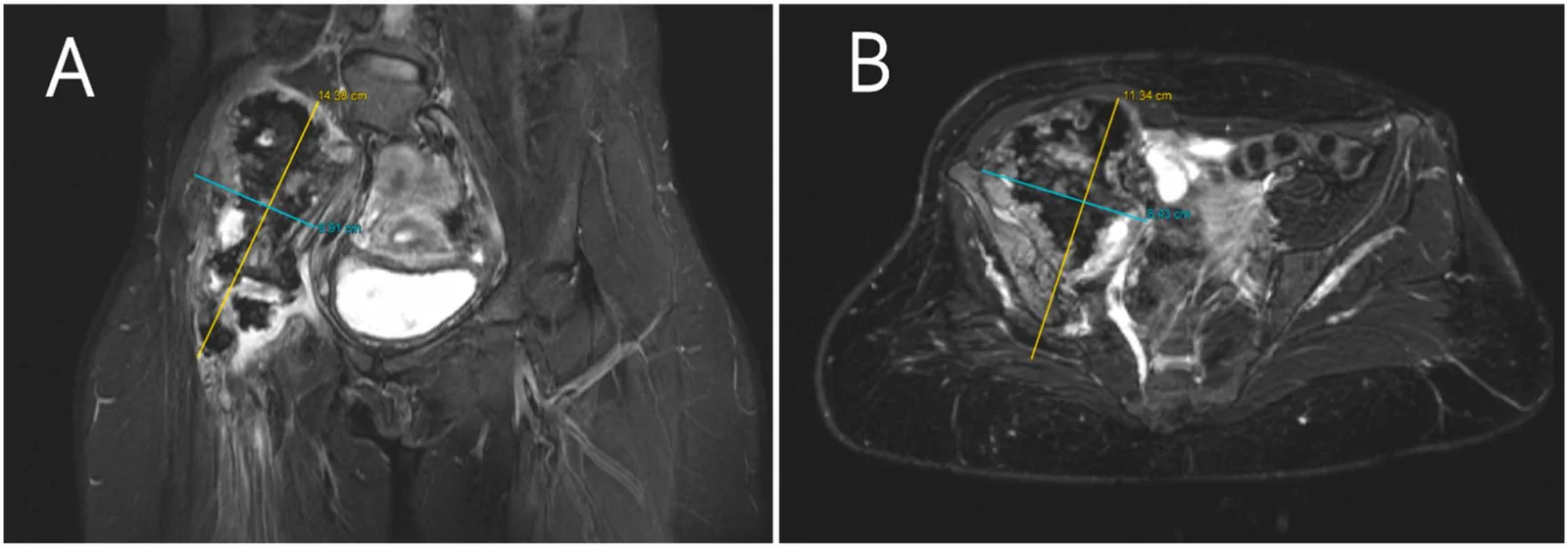
MRI of the pelvis from 14 February 2025. Considering the different examination technique, largely constant-size, partially calcified brucellosis manifestation with infiltration of the right ileal os. Furthermore, there is new formation suspicious for abscess, which runs ventrally from the central parts of the brucellosis manifestation, extends into the right-sided abdominal musculature and leads to inflammatory involvement of the neighboring soft tissues. Also shown is the constant presentation of the two known fistula tracts, starting from the brucellosis manifestation. A. Coronal reconstruction, B. Axial reconstruction.

By April 2025, the patient remained clinically stable and fever-free, although she continued to report pain in the right hip region due to exertion. Antibiotic therapy with rifampicin and doxycycline was continued while the results of the cultures from the reference laboratory were still pending. Multiple PCR tests for *Brucella spp.* DNA from the fistula secretions were negative, but the fistula formation persisted. In addition, serological testing subsequently showed falling antibody concentrations (Table 2).

**Table 2:** Serological detection.

Examination	07/2018	11/2021	02/2023	01/2025	07/2025	*Reference*
*Brucella spp.* IgG - ELISA [U/ml]	475	4102	3798	1639	451	*< 20 U/ml*
*Brucella spp.* IgM - ELISA [U/ml]	< 15	< 15	< 15	< 15	< 15	*< 15 U/ml*
*Brucella spp.* Antibodies - Brucellacapt	1:1280	1:2560	1:1280	1:640	*N/A*	*< 1:40*

## Discussion

In this case, we report a rare presentation of chronic brucellosis with persistent, calcified intraosseous and soft tissue lesions, complicated by recurrent fistula formation and intermittent reactivation over a period of nearly seven years. While osteoarticular involvement is a known complication of chronic brucellosis, the extensive intrapelvic mass with ossifying features, prolonged disease course, and repeated fistula formation observed in our patient are highly uncommon. The differential diagnosis for granulomatous bone lesions with caseous necrosis is broad and includes tuberculosis, sarcoidosis, and fungal infections. However, in patients with epidemiological risk factors—such as migration from endemic areas—brucellosis must be considered [1]. In our case the diagnosis was confirmed by culture, PCR, and histopathology. Crucially, serial negative PCRs from fistula drainage in the advanced phase did not rule out persistent infection. PCR, when combined with serology, showed to be a useful additional diagnostic test for suspected cases and relapses of brucellosis [5], [6].

It can be assumed that the patient's infection took place several years before the first clinical manifestation due to the significantly higher incidence in Somalia. Notably, reactivations of brucellosis have been documented even decades after initial exposure [7].

The clinical presentation and therapy regimen are most comparable to the more common vertebral osteomyelitis in brucellosis. There are larger case series reporting brucellar spondylitis and osteomyelitis with soft tissue abscesses, some of which mimicked malignancy or tuberculosis due to granulomatous inflammation with caseous necrosis [8], [9]. In a large study involving 96 patients with brucellar vertebral osteomyelitis two treatment strategies were compared: a DOX-STR regimen (doxycycline for three months plus streptomycin for 2–3 weeks) and a DOX-RIF regimen (doxycycline plus rifampicin, both for three months) [8]. No significant differences were observed between the two groups regarding treatment failure, mortality, or relapse [8]. However, the study underscored the necessity of prolonged therapy to ensure eradication of infection, particularly in patients with deep-seated lesions. Compared to our case, the standard DOX-RIF regimen was similarly applied as long-term oral therapy. However, due to the presence of deep-seated ossified lesions and recurrent fistula formation, intravenous gentamicin was added repeatedly as a third agent to enhance bactericidal effect. Our patient required multiple treatment cycles, including a prolonged 29-day initial gentamicin course due to subtherapeutic serum levels. Additionally, therapy had to be paused and reinitiated several times due to side effects and poor adherence, which likely contributed to persistent infection. Nevertheless, the patient completed multiple in-hospital treatment regimens, which in total should have led to effective treatment. Furthermore, no evidence of antimicrobial resistance was identified despite targeted microbiological investigations.

A possible explanation for the prolonged disease course was found in in-vitro tests with *Brucella abortus*. While the exact mechanism is still unclear, it has been shown that *Brucella spp.* are able to form “persister” cells within macrophages. These bacterial subpopulations exhibit increased tolerance to antimicrobial treatment and may survive under adverse conditions. Following cessation of therapy, they may regain metabolic activity and potentially contribute to recurrent infection [10]. Moreover, *in vitro* studies have shown that *Brucella abortus* can infect and replicate within osteoblasts, where it impairs bone formation by inhibiting mineral and organic matrix deposition, induces osteoblast apoptosis, and promotes receptor activator of nuclear factor κB ligand (RANKL) expression, thereby favoring bone resorption. Furthermore, infected macrophages release pro-inflammatory mediators, including tumor necrosis factor alpha (TNF-α), which may indirectly impair osteoblast survival and function [11]. However, the clinical relevance of this phenomenon in human brucellosis remains incompletely understood, and other factors such as limited antibiotic penetration into calcified lesions may also have contributed to the persistent disease course observed in this case.

Furthermore, repeatedly negative PCR results do not necessarily exclude persistent focal infection. In chronic brucellosis, bacterial loads may become extremely low due to intracellular localization, fibrosis, and calcification of infected tissue. The diagnostic performance of PCR is therefore highly dependent on sampling site and specimen quality. Previous studies demonstrated that PCR can detect as little as DNA equivalent to approximately 2–20 bacterial cells; however, focal lesions may contain unevenly distributed bacterial populations, increasing the risk of sampling error and false-negative results [12].

The contribution of antimicrobial resistance to the prolonged disease course remains uncertain. While antimicrobial susceptibility testing of the initial *Brucella melitensis* isolate showed no evidence of resistance (Table 1), no further resistance testing could be performed during follow-up because the pathogen was no longer cultivable and only intermittent PCR positivity was observed.

The role of surgery in chronic osteoarticular brucellosis is generally limited and must be considered on an individual basis. In our patient, surgical procedures were primarily performed for diagnostic confirmation and microbiological sampling. Although the lesion was extensive and associated with persistent fistula formation, repeated histopathological examinations showed no evidence of malignancy. Given the complex intrapelvic extension of the lesion and the expected morbidity of major pelvic surgery, radical resection was not considered feasible. Therefore, a conservative approach with prolonged antimicrobial therapy and close follow-up was favored. As is commonly known, the mortality rate in brucellosis is predominantly associated with endocardial involvement [13], [14], [15]. No cardiac complications were observed in our patient. However, the disease's chronic course, which included recurrent pain, fistulas, and exhaustion, resulted in a significant and protracted decline in quality of life.

## Conclusion

This case illustrates the diagnostic and therapeutic challenges of chronic brucellosis with ossifying lesions. Despite repeated and prolonged combination therapies with multiple antibiotics, complete eradication of the infection was not achieved. The clinical stability, alongside persistent fistula formation and exertion-related pain, indicates ongoing low-grade inflammatory activity. Several limitations should be acknowledged. Microbiological confirmation during follow-up was limited because *Brucella melitensis* could only be cultured at initial diagnosis, while subsequent assessments relied mainly on PCR and imaging findings. Consequently, repeat antimicrobial susceptibility testing was not possible. Furthermore, the exact mechanism underlying the prolonged disease course remains uncertain and is likely multifactorial, involving factors such as incomplete adherence, limited antibiotic penetration into calcified lesions, and bacterial persistence. Nevertheless, this case provides important clinical lessons. Chronic brucellosis should be considered in the differential diagnosis of granulomatous bone lesions, particularly in patients originating from endemic regions. In addition, negative PCR results do not necessarily exclude persistent focal infection, especially in calcified or ossified lesions. This case underscores the need for individualized, multidisciplinary care and further research into diagnostic tools and optimal treatment strategies for chronic brucellosis.

## CRediT authorship contribution statement

**Christoph Zacher:** Writing – original draft, Visualization, Conceptualization. **Sebastian Dolff:** Writing – review & editing, Supervision. **Oliver Witzke:** Writing – review & editing, Supervision. **Lukas van de Sand:** Writing – original draft, Visualization, Conceptualization.

## Consent to participate and publish

The patient reported in this case has given us her explicit consent for publication and the use of her health data.

## Funding

This research received no external funding. Oliver Witzke is supported by an unrestricted grant from the 10.13039/501100014837Rudolf-Ackermann-Stiftung (Stiftung für Klinische Infektiologie).

## Declaration of Competing Interest

The authors have no relevant financial or non-financial interests to disclose related to this study. Oliver Witzke has received research funding, speaker honoraria, or travel support from the following companies: Amgen, Alexion, Astellas, AstraZeneca, Basilea, Biotest, Bristol-Myers Squibb, Correvio, Chiesi, Gilead, GSK, Hexal, Janssen, Dr. F. Köhler Chemie, MSD, Novartis, Roche, Pfizer, Sanofi, TEVA, and UCB.

## Data Availability

The original contributions presented in the study are included in the article. Further inquiries can be directed to the corresponding author.
